# The impact of mental fatigue on brain activity: a comparative study both in resting state and task state using EEG

**DOI:** 10.1186/s12868-020-00569-1

**Published:** 2020-05-12

**Authors:** Gang Li, Shan Huang, Wanxiu Xu, Weidong Jiao, Yonghua Jiang, Zhao Gao, Jianhua Zhang

**Affiliations:** 1 College of Engineering, Zhejiang Normal University, 688 Yingbin Road, Zhejiang 321004 Jinhua, People’s Republic of China; 2 Key Laboratory of High Efficiency and Clean Mechanical Manufacture, Ministry of Education of China, School of Mechanical Engineering, Shandong University, 688 Yingbin Road, Zhejiang 321004 Jinan, People’s Republic of China

**Keywords:** Mental fatigue, EEG, Resting state, Task state

## Abstract

**Background:**

Mental fatigue is usually caused by long-term cognitive activities, mainly manifested as drowsiness, difficulty in concentrating, decreased alertness, disordered thinking, slow reaction, lethargy, reduced work efficiency, error-prone and so on. Mental fatigue has become a widespread sub-health condition, and has a serious impact on the cognitive function of the brain. However, seldom studies investigate the differences of mental fatigue on electrophysiological activity both in resting state and task state at the same time. Here, twenty healthy male participants were recruited to do a consecutive mental arithmetic tasks for mental fatigue induction, and electroencephalogram (EEG) data were collected before and after each tasks. The power and relative power of five EEG rhythms both in resting state and task state were analyzed statistically.

**Results:**

The results of brain topographies and statistical analysis indicated that mental arithmetic task can successfully induce mental fatigue in the enrolled subjects. The relative power index was more sensitive than the power index in response to mental fatigue, and the relative power for assessing mental fatigue was better in resting state than in task state. Furthermore, we found that it is of great physiological significance to divide alpha frequency band into alpha1 band and alpha2 band in fatigue related studies, and at the same time improve the statistical differences of sub-bands.

**Conclusions:**

Our current results suggested that the brain activity in mental fatigue state has great differences in resting state and task state, and it is imperative to select the appropriate state in EEG data acquisition and divide alpha band into alpha1 and alpha2 bands in mental fatigue related researches.

## Background

Mental fatigue refers to a condition of low alertness and cognitive impairment [1]. Too much brain activity and stimulation can make a person feel mentally exhausted, and this feeling is akin to physical fatigue. Mental fatigue can give rise to numerous bad consequences, for example, making the uncomplicated tasks turn to be increasingly difficult or even impossible. Mental fatigue has become a popular sub-healthy state in nowadays, which has effects on almost all aspects of cognitive functions of human brain [2], such as driving fatigue [3]. Considering the impacts of mental fatigue on our daily life, it is very important to reveal the differences of mental fatigue on brain activity in resting state and task state at the same time.

Previous studies have been centered on the changes associated with task-related brain activity [4, 5]. The fatigue-inducing mental tasks were widely used by researchers. The mental tasks that requiring different intensity of attention can differentiate the levels of mental fatigue, and their experimental duration could be distinct. The *n*-back task [6, 7] and psychomotor vigilance task [8, 9] (PVT) can be categorized as high-attention-demanding tasks, sleep deprivation [10] can be classified into low-attention-demanding tasks, meanwhile mental arithmetic task [11] and driving simulation task [12] fall into middle-attention-demanding tasks. However, among these three-type tasks, the middle-attention-demanding tasks are greatly in line with our daily working load. Therefore, we chose mental arithmetic task to induce mental fatigue.

Historically, mental fatigue has been most prevalently studied with the neuroimaging technique of EEG [13–15]. It has been widely proved that mental fatigue can result in obvious changes in EEG signals [16]. Strijkstra has found that EEG at resting state shows strong negative correlations of alpha power and positive correlations of theta power with subjective sleepiness [17]. With the mental fatigue increasing, the power of alpha rhythm increases when the eyes are open and decreases when the eyes are closed [18]. These changes in EEG can be used to detect mental fatigue [11, 16], which is especially important and meaningful for driving fatigue estimation [8, 12]. From the above, we can conclude that EEG has become the most effective technical means for exploring the neuromechanism and detection of mental fatigue [19, 20].

The current study is also motivated by the studies which have divided the EEG bands into narrower bands. In mental fatigue related studies, some researchers divided alpha band into alpha1 (8–10 Hz) and alpha2 (10–13 Hz). Li has performed statistical analysis on the characteristics of alpha1 and alpha2 to estimate mental fatigue, and reported that alpha1 band is better for fatigue detection [11]. Sun has applied alpha1 frequency band for mental fatigue classification, and achieved a high prediction accuracy [21].

In this study, we would explore the differences of mental fatigue on electrophysiological activity both in resting and task states at the same time. To this end, we administered a group of challenging sustained mental arithmetic math tasks for mental fatigue induction among the recruited young healthy male participants, and EEG data for resting and task states before and after each task segments were collected. Then the power and relative power of delta, theta, alpha1, alpha2, and beta were computed, and statistical analysis were carried on the results among different brain regions.

## Results

**Fig. 1:**
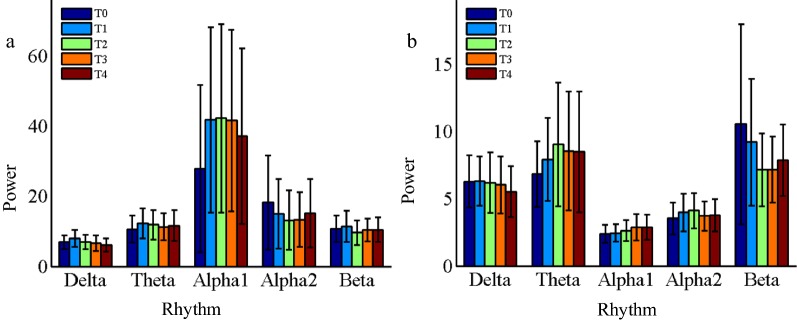
The power of EEG rhythms over the whole brain region at different time periods. **a** Resting state, **b** Task state

Figure 1 and Table 1 depict the results of EEG power. It has been found that the power of each rhythm in resting state and task state varies in the evolutionary process of mental fatigue, but there are few statistical differences and FDR correction are not performed on the results of the *P*-values. In resting state, the alpha1 rhythm power at frontal and temporal regions have a marked tesndency to increase, and the alpha2 power at parietal region has a significant reduction. In task state, only the beta power at parietal and occipital regions have obvious decrease. The reason for the insignificant statistical differences may be that the power of EEG rhythms is not sensitive to the mental fatigue, and the small changes of EEG power will be masked by individual differences and power spectrum fluctuations. Therefore, the other widely used EEG index, relative power, was analyzed in the study.

**Table 1:** ANOVA results of *P*-value for EEG power in different brain regions in resting state and task state

State	Region	Delta	Theta	Alpha1	Alpha2	Beta
Resting state	Whole	0.118	0.792	0.403	0.572	0.710
	Frontal	0.366	0.819	*0.044*	0.372	0.656
	Temporal	0.135	0.574	*0.011*	0.221	0.078
	Central	0.513	0.887	0.119	0.114	0.273
	Parietal	0.141	0.735	0.203	*4.3E − 4*	0.112
	Occipital	0.492	0.796	0.914	0.159	0.089
Task state	Whole	0.760	0.507	0.212	0.673	0.103
	Frontal	0.833	0.728	0.735	0.390	0.684
	Temporal	0.106	0.179	0.765	0.903	0.193
	Central	0.965	0.845	0.434	0.743	0.195
	Parietal	0.644	0.426	0.059	0.057	*8.9E − 7*
	Occipital	0.223	0.724	0.757	0.324	*0.013*

**Fig. 2:**
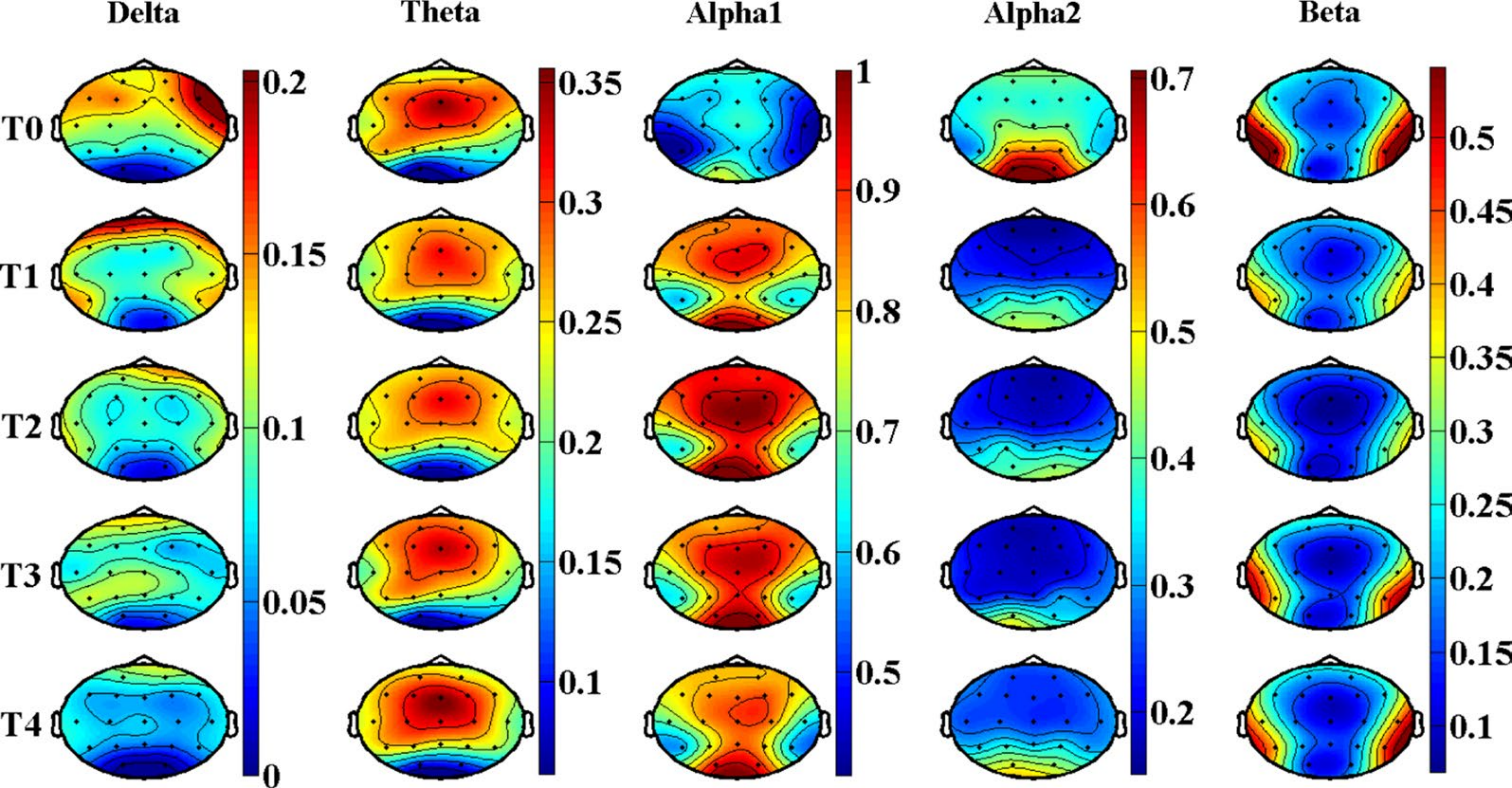
Brain topography of the relative power of EEG rhythms in resting state. In the figure, all the relative power values are normalized to 0–1

**Fig. 3:**
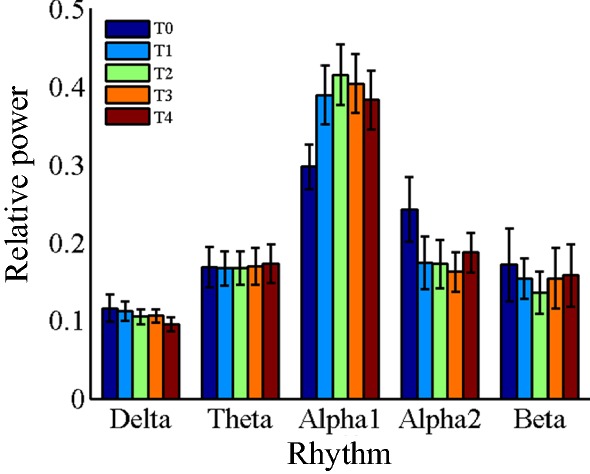
The average relative power of EEG rhythms over the whole brain region at different time periods in resting state

Figures 2 and 3, Tables 2, and 3 show the results of EEG relative power in resting state. Figure 2 is the brain topography of the relative power for every rhythms. Figure 3 is the average relative power of all electrodes. Table 2 is the statistical results of *P* value, *F*-value and $\eta_{p}^{2}$ for EEG relative power in different brain regions, which shows very good statistical differences according to the results of *P*-value, *F*-value and $\eta_{p}^{2}$. Therefore, FDR correction was performed on the results of *P*-value among different brain regions to reduce the risk of false positive. Table 3 is the corrected statistical differences corresponding to Table 2. As shown in Figs. 2 and 3, Tables 2 and 3, the relative power of delta rhythm presents a monotonically decreasing trend throughout the whole brain, and has corrected significant statistical differences across all brain regions. The results of theta rhythm indicate that only in the central region has a corrected significant increasing trend [*P *= 0.005, *F *= 7.29, $\eta_{p}^{2}$ = 0.74, corrected *P *= 0.03], and there are no statistic difference in other brain regions. Whereas, both alpha1 rhythm and alpha2 rhythm have corrected significant statistical differences among all the brain regions, and beta rhythm have corrected significant statistical differences only in temporal region [*P *= 7.3E − 5, *F *= 13.5, $\eta_{p}^{2}$ = 0.78, corrected *P *= 4.4E − 4]. Besides, the changing regularities of alpha1, alpha2, and beta rhythms are not monotonous.

**Table 2:** ANOVA results of *P*-value, *F*-value and $\eta_{p}^{2}$ for relative power EEG rhythms in different brain regions in resting state

Region	Delta			Theta			Alpha1			Alpha2			Beta		
	*P*	*F*	$\eta_{p}^{2}$	*P*	*F*	$\eta_{p}^{2}$	*P*	*F*	$\eta_{p}^{2}$	*P*	*F*	$\eta_{p}^{2}$	*P*	*F*	$\eta_{p}^{2}$
Whole	*1.5E − 5*	7.98	0.26	0.955	0.17	0.01	*1.4E − 15*	29.3	0.57	*7.1E − 11*	18.0	0.44	0.079	2.16	0.09
Frontal	*2.2E − 5*	10.3	0.58	0.664	0.60	0.07	*3.4E − 15*	75.2	0.91	*4.5E − 20*	168	0.96	*0.038*	2.91	0.28
Temporal	*0.025*	3.81	0.50	0.727	0.51	0.12	*2.3E − 4*	10.9	0.74	*0.012*	4.72	0.56	*7.3E − 5*	13.5	0.78
Central	*2.0E − 4*	16.5	0.87	*0.005*	7.29	0.74	*1.7E − 6*	47.9	0.95	*2.1E − 9*	193	0.99	0.117	2.42	0.09
Parietal	*1.8E − 4*	17.1	0.86	0.309	1.38	0.06	*5.5E − 4*	13.1	0.84	*1.2E − 5*	31.7	0.93	*0.043*	3.68	0.59
Occipital	*0.015*	9.42	0.89	0.950	0.16	0.03	*0.004*	16.4	0.93	*0.002*	22.3	0.95	0.750	0.48	0.02

Statistically significant differences are highlighted in italics (*P* < 0.05)

**Table 3:** Corrected results of *P*-value corresponding to Table 2 among different brain

Region	Delta	Theta	Alpha1	Alpha2	Beta
Whole	*6.6E − 5*	0.955	*8.4E − 15*	*2.1E − 10*	0.1185
Frontal	*6.6E − 5*	0.955	*1.0E − 14*	*2.7E − 19*	0.086
Temporal	*0.025*	0.955	*3.5E − 5*	*0.012*	*4.4E − 4*
Central	*3.0E − 4*	*0.030*	*3.4E − 6*	*4.2E − 9*	0.1404
Parietal	*3.0E − 4*	0.927	*6.6E − 4*	*1.8E − 5*	0.086
Occipital	*0.018*	0.955	*0.004*	*2.4E − 3*	0.75

Statistically significant differences are highlighted in italics (*P* < 0.05)

**Fig. 4:**
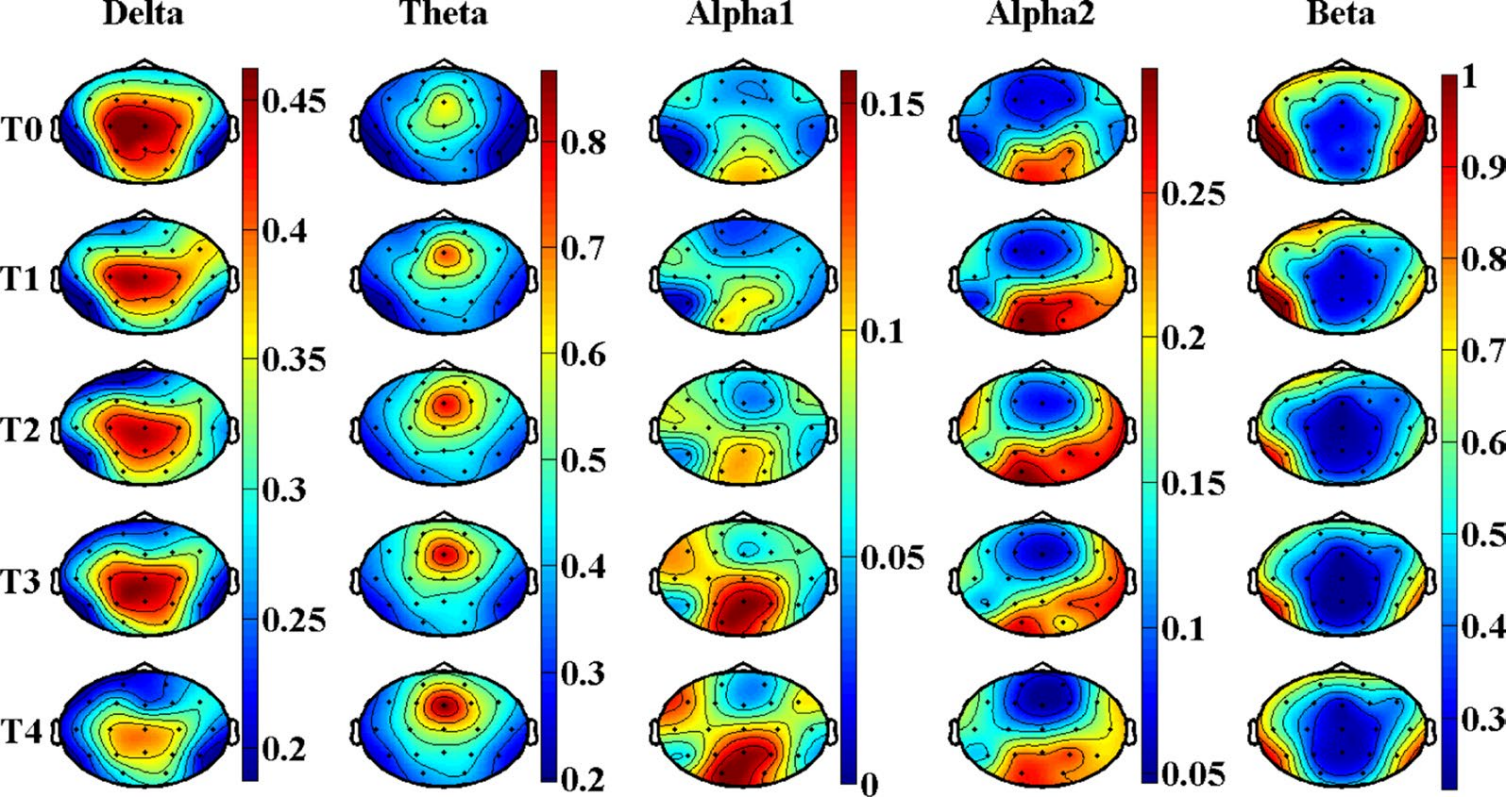
Brain topography of the relative power of EEG rhythms in task state. In the figure, all the relative power values are normalized to 0–1

**Fig. 5:**
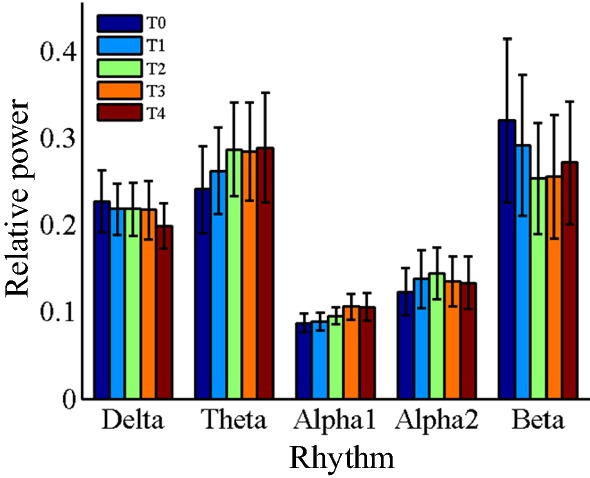
The average relative power of EEG rhythms over the whole brain region at different time periods in task state

Figures 4 and 5, Tables 4 and 5 show the results of EEG relative power in task state. Figure 4 is the brain topography of the relative power for every rhythms, and Fig. 5 is the average relative power of all electrodes. Table 4 is the statistical results of *P*-value, *F*-value and $\eta_{p}^{2}$ for EEG relative power in different brain regions. Table 5 is the corrected statistical differences corresponding to Table 4 obtained by FDR correction performed on the results of *P*-value among different brain regions. As shown in Figs. 4 and 5, Tables 4 and 5, the relative power of delta rhythm presents a decreasing trend throughout the whole brain, and has corrected significant statistical differences in frontal region [*P *= 0.003, *F *= 5.22, $\eta_{p}^{2}$ = 0.41, corrected *P *= 0.012], central region [*P *= 0.004, *F *= 7.69, $\eta_{p}^{2}$ = 0.76, corrected *P *= 0.012], and parietal region [*P *= 0.006, *F *= 7.02, $\eta_{p}^{2}$ = 0.74, corrected *P *= 0.012]. Both theta rhythm and alpha1 rhythm have a non-monotonic increasing trend for the results of relative power, but only in temporal region for theta rhythm [*P *= 0.001, *F *= 7.67, $\eta_{p}^{2}$ = 0.67, corrected *P *= 0.006], and only in central region [*P *= 7.7E − 5, *F *= 20.8, $\eta_{p}^{2}$ = 0.89, corrected *P *= 4.6E − 4] and parietal region [*P *= 3.6E − 4, *F *= 14.5, $\eta_{p}^{2}$ = 0.85, corrected *P *= 0.001] for alpha1 rhythm that have corrected significant statistical differences. As for alpha2 rhythm and beta rhythm, no corrected statistically significant differences are observed.

**Table 4:** ANOVA results of *P*-value, *F*-value and $\eta_{p}^{2}$ for relative power EEG rhythms in different brain regions in task state

Region	Delta			Theta				Alpha1			Alpha2			Beta		
	*P*	*F*	$\eta_{p}^{2}$	*P*	*F*	$\eta_{p}^{2}$	*P*		*F*	$\eta_{p}^{2}$	*P*	*F*	$\eta_{p}^{2}$	*P*	*F*	$\eta_{p}^{2}$
Whole	0.109	1.95	0.08	*0.044*	2.56	0.10		0.056	8.99	0.29	0.311	1.21	0.05	0.060	2.53	0.09
Frontal	*0.003*	5.22	0.41	0.114	2.04	0.11		0.115	3.68	0.33	0.526	0.81	0.09	0.197	1.62	0.11
Temporal	0.406	1.07	0.02	*0.001*	7.67	0.67		0.225	3.80	0.50	0.084	2.53	0.4	*0.015*	4.41	0.54
Central	*0.004*	7.69	0.76	0.424	1.06	0.02		*7.7E − 5*	20.8	0.89	0.859	0.32	0.01	0.326	1.32	0.05
Parietal	*0.006*	7.02	0.74	*0.021*	4.72	0.65		*3.6E − 4*	14.5	0.85	0.164	2.04	0.12	0.103	8.25	0.07
Occipital	*0.037*	6.03	0.83	0.579	0.79	0.03		0.076	4.12	0.27	0.540	0.87	0.02	0.238	2.03	0.13

Statistically significant differences are highlighted in italics (*P* < 0.05)

**Table 5:** Corrected results of *P*-value corresponding to Table 4 among different brain

Region	Delta	Theta	Alpha1	Alpha2	Beta
Whole	0.131	0.088	0.112	0.622	0.180
Frontal	*0.012*	0.171	0.138	0.648	0.286
Temporal	0.406	*0.006*	0.225	0.492	0.090
Central	*0.012*	0.509	*4.6E − 4*	0.859	0.326
Parietal	*0.012*	0.063	*0.001*	0.492	0.206
Occipital	0.056	0.579	0.114	0.648	0.286

Statistically significant differences are highlighted in italics (*P* < 0.05)

## Discussion

In the present study, we analyzed the difference in spontaneous neural activities caused by performing prolonged fatigue-inducing mental arithmetic tasks both in resting state and task state. Five EEG rhythms were evaluated among five brain regions in the two states. The delta rhythm power was 7.1 ± 0.54 μV^2^ in the resting state, and had the lowest proportion (10%) in all EEG rhythms; in the task state, the power was 6.1 ± 0.34 μV^2^, but the proportion increased to 21.5%. This is mainly because Alpha1 and Alpha2 rhythms were significantly suppressed in task state (see Fig. 4), leading to a significant increase in the proportion of corresponding delta rhythm. The delta rhythm power had no statistical difference both in resting and task states (see Table 6), which is in line with its actual physiological meaning. Because delta rhythm is related to people’s deep sleep [22], and it usually appears in large quantities in adult’s deep sleep, anesthesia and hypoxia. As for the relative power of delta rhythm, it decreased significantly along with the accumulation of task time, which is consistent with the results reported by Jap when researching driving fatigue [23]. Some literatures have also pointed out that the amplitude and relative power of delta rhythm increased under fatigue state [24]. However, in many fatigue evaluation studies, delta band was directly removed by researchers and technicians [22]. Because they believe that delta rhythm reflects the state of deep sleep, and general brain fatigue status does not show significant changes. Moreover, the frequencies of EEG artifacts (such as blink artifacts, eye movement artifacts, electrocardio artifacts, etc., except for power–frequency artifacts and myoelectricity artifacts) mainly coincide with the delta frequency band. The removal of the artifacts is highly subjective, and the removal effect varies from person to person. Therefore, the results of delta rhythm in this study will not be in further discussion.

**Table 6:** Division of the brain regions and their included electrode

Brain region	Electrode name
Frontal	Fp1, Fp2, F3, F4, F7, F8, Fz
Temporal	T3, T4, T5, T6
Central	C3, Cz, C4
Parietal	P3, Pz, P4
Occipital	O1, O2

The power and relative power results of theta rhythm were unanimous both in resting state and task state, demonstrating an increasing trend, which was consistent with the results of most fatigue studies [17, 22–24]. Generally, theta rhythm is considered to reflect the early state of sleepiness [25], which is related to brain fatigue [26] and has a sensitive response to fatigue [27]. As shown in Tables 3 and 5, the response results of theta rhythm in the task state were slightly better than that in the resting state, because there were corrected statistical differences in the temporal region and parietal region in the task state, while there were corrected statistical differences only in the central region in the resting state.

Alpha rhythm reflects the state of relaxation and wakefulness. When focusing attention, external stimulation or visual input, alpha rhythm will be blocked [28]. Alpha rhythm is considered to be the most sensitive indicator of brain fatigue [26, 27], which is consistent with the statistical analysis results of alpha1 and alpha2 showed in Tables 2, 3, 4, and 5. Along with the increase of mental fatigue, the power and relative power of alpha rhythm were reported to be significantly increased [22, 24]. Several other researchers reported the opposite changing tendency [23]. However, it is now widely accepted that alpha rhythm intensifies as the brain transformed from normal into fatigue [29, 30] (see detailed statistical results of relevant studies in Ref. [27]). As shown in Tables 3 and 5, the effect of alpha1 and alpha2 rhythm in depicting mental fatigue in resting state is better than that in task state.

In this study, alpha band was further divided into two sub-bands, alpha1 band and alpha2 band, obtaining some meaningful results: the relative power of alpha1 rhythm increased significantly both in resting state and task state, while alpha2 rhythm decreased significantly in the resting state, but showed an increasing trend in the task state, which were consistent with the power change trends shown in Fig. 1. In similar research results, it is also pointed out that alpha1 rhythm power increases with the increase of fatigue level [21, 30, 31], and alpha2 rhythm has the same changes in task state [30]. The changing regularities of alpha1 and alpha2 in the resting state is completely opposite, and that in the task state is consistent, indicating that it is essential to divide alpha frequency band into alpha1 and alpha2 sub-bands in brain fatigue research based on EEG. Klimesch has emphasized that using narrower frequency bands in the study can reduce the risk that the frequency effects are cancelled out or not discovered [32], which is well demonstrated in the results of alpha1 and alpha2 rhythms in this study. In addition, narrower frequency band division can enhance the physiological meaning of the sub-bands and make their statistical results more significant. The contrary changing trend and significant statistical results of Alpha1 and Alpha2 rhythms in the resting state can prove this inference.

Klimesch has pointed out through the analysis of event-related potential that alpha1 rhythm is related to attention, and its power will increase significantly when the attention task increases and the subjects are required to stay awake and not allow sleep and rest [32], which is consistent with the results of alpha1 rhythm in this study. As for alpha2 rhythm, Klimesch et al. have indicated that alpha2 desynchronization is positively correlated with brain long-term memory function by comparing the performance of subjects with different memory abilities in memory tasks [33]. In their subsequent studies, it has been further proved that alpha2 rhythm is correlated with memory [34–37] and cognitive behavior [38]. When the memory task increases, alpha2 rhythm (in the state of eye closure at the time of EEG data collection) shows synchronization [34, 35, 39], that is, the power decreases, which can explain the changing trend of alpha2 rhythm in the resting state in this study.

Further analysis of the brain topography in the fourth column of Figs. 2 and 4, we found that: (a) alpha2 rhythm power is mainly distributed in the occipital region, which is consistent with the results of topography given by Craig et al. [30]; (b) in resting state, alpha2 rhythm is very strong in all brain regions in the baseline state (referring to the T0 period), but when the brain enters into the fatigue state (referring to the T1, T2, T3 and T4 periods), alpha2 rhythm is mainly concentrated in the occipital region; (c) in task state, alpha2 rhythm is mainly concentrated in occipital region, and tends to strengthen in the parietal region and right temporal region with the increase of tasks. The above results suggest that alpha2 rhythm is also closely related to visual information processing in the brain, as the occipital lobe is mainly responsible for visual functions. In the resting state, there is no visual information input in the brain, and the influence of memory task may be dominant in the brain, so the brain is shown as de-synchronization [33], and the power and relative power are shown as decreased. In the task state, the brain has a large amount of visual information to be processed, then the neural centers in the occipital area and nearby brain areas are activated (manifested as increased energy of alpha2 rhythm) to complete the visual information transmission and processing tasks. The influence of visual information processing task is dominant, while the influence of memory task is covered. Under the combined action of these two comprehensive effects, the power of alpha2 rhythm has increasing trend, but no statistical difference.

With the deepening of mental fatigue, the relative power of beta rhythm decreases significantly both in resting state and task state, which is consistent with the change trend of its power. Consistent research results have also been widely reported [22, 23]. Beta rhythm is usually associated with the excited state of the brain (e.g., mood and mental activity). When the brain converts from resting state to task state, it needs to maintain a high concentration to complete the tasks, and its beta proportion rises from 15% to 28%. According to the brain topography in the fifth column of Figs. 2 and 4, beta rhythm is mainly distributed in the temporal region, which is consistent with the results of the brain topography given by Jap et al. [23]. Based on the statistical results in Tables 3 and 5, the effect of beta rhythm on depicting mental fatigue in resting state is slightly better than that in task state.

## Conclusions

In this study, we attempted to study the differences of mental fatigue on electrophysiological activity both in resting and task states at the same time. A group of mental arithmetic math problems was performed for mental fatigue induction. EEG data was collected before and after the tasks. Then five EEG rhythms (delta, theta, alpha1, alpha2, and beta) were calculated and discussed both in resting and task states. The results suggested the following conclusions: firstly, the task of mental arithmetic problems can successfully induce mental fatigue in the enrolled subjects; secondly, the relative power index of each EEG rhythm is more sensitive than the power index in response to mental fatigue, suggesting that relative power can be applied to estimate brain fatigue level; thirdly, the relative power of each EEG rhythm is better at assessing mental fatigue in resting state than in task state; finally, it is of great physiological significance to divide alpha frequency band into alpha1 band and alpha2 band in fatigue related studies, and at the same time improve the statistical differences of sub-bands.

## Methods

### Participants

In this study, 20 right-handed and healthy male participants of engineering postgraduate students (age: 24.5 ± 1.5 years, BMI: 20.7 ± 1.8 kg/m^2^) were recruited. Each subject must have regular living habits and normal eyesight, and have no brain disorders. All participants were asked to follow the bellow requirements before the tests: (1) forbid to stay up late and drink alcohol and drugs within a week, (2) prohibit smoke, coffee and tea within 8 h, and (3) wash the hair two hours ago. Every participant signed the informed consent, and Shandong University Ethics Committee approved our study. Detailed descriptions about the participants were introduced in the Ref. [40].

### EEG data recording and preprocessing

A mental fatigue model was carried out to induce fatigue among all recruited participants: do two hundred different mental arithmetic problems (one number between sixty and ninety plus another number between sixty and ninety, and then multiplied by a number between six and nine) for one hundred minutes. All problems were strictly designed to be at appropriate difficulty level and can be finished within thirty-seconds according to preceding pretests. That is, all the participants can get high accuracies during the divided four tasks. The results of the accuracies were similar and had no statistic difference among these four tasks. What we concerned was the effects of tasks on the brain when the participants highly focused on the given mental arithmetic math tasks. Detailed descriptions about the experimental design were introduced in the Ref. [40].

**Fig. 6:**
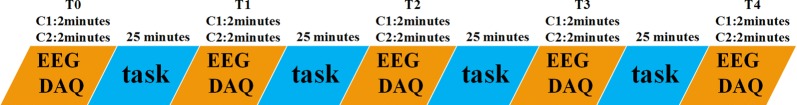
EEG data acquisition (EEG DAQ) procedures. C1 means resting state and C2 means task state

As depicted in Fig. 6, these two hundred problems were evenly divided into four task segments, and 19-channel EEG data were recorded before and after these four task segments for both resting state and task state (2 minutes EEG data recordings for each state). Thus, five times EEG recordings named as T0, T1, T2, T3 and T4 were implemented in total. Then, 10 pieces of sequential 5-second EEG signals with no artifacts were selected by EEGLAB for each state (Only eighteen participants’ EEG signals were further analyzed, because the other two were excluded due to their large head movements during EEG collections). EEG rhythms of delta (2–4 Hz), theta (4–8 Hz), alpha1 (8–10 Hz), alpha2 (10–13 Hz), and beta (13–30 Hz) were extracted by digital FFT filtering. Detailed descriptions and definitions about EEG data recordings and preprocessing were introduced in the Ref. [40].

### Computation of EEG indices

In this study, the power and relative power of every EEG rhythms were explored. The frequency spectrum *X*(*f*) of EEG signal *x*(*n*) was obtained by means of FFT, and then the power spectrum *P*_*x*_(*f*) of EEG were gained with Eq. (1). The power *E*(*h*) and relative power *R*(*h*) were calculated through Eq. (2) and Eq. (3). Where, in Eq. (1), Eq. (2) and Eq. (3), *N* is the number of EEG signal *x*(*n*), *h* represents the EEG rhythms (such as delta, theta, alpha1, alpha2, beta), *f*_*h*_ and *f*_*l*_ are the upper and lower frequencies of *h* rhythm respectively, *E*_*total*_ is the total power of all EEG rhythms. Besides, all the calculated power spectrum are the average value of the selected 10-segment EEG signals for each condition. In order to study the differences of mental fatigue in different brain regions [23], we divided the whole brain region into five brain functional regions. As shown in Table 6, the nineteen electrodes are also divided into five groups. And EEG indices are also computed based on the five brain functional regions.

1 $$ P_{x} (f) = \frac{1}{N}\left| {X(f)} \right|^{2} . $$

2 $$ E(h) = \frac{1}{{f_{h} - f_{l} }}\int_{{f_{l} }}^{{f_{h} }} {P_{x} (f)} df. $$

3 $$ R(h) = \frac{E(h)}{{E_{total} }}. $$

### Statistical analysis

One-way analysis of variance (ANOVA) was performed on the power and relative power of EEG bands to distinguish the statistically significant differences among the five periods. *P*-value, *F*-value and $\eta_{p}^{2}$ (partial eta squared) are given in the ANOVA results. In order to make the statistical results more convincing and reduce the risk of false positive, FDR (false discovery rate) correction was carried out for the *P*-values among different brain regions.

## Data Availability

The datasets used and/or analyzed during the current study are available from the corresponding author on reasonable request.

## Abbreviations

EEG: Electroencephalogram
ANOVA: Analysis of variance
FDR: False discovery rate
EEG DAQ: EEG data acquisition
